# Plasma Lipidomics Identify Pathways Linked to Acute Lung Injury in a Porcine One-Lung Ventilation Surgery Model

**DOI:** 10.3390/ijms27125219

**Published:** 2026-06-09

**Authors:** Simone C. da Silva Rosa, Evan Gauvin, Dagem Chernet, Jay Kormish, Catherine Giffin, Martha Hinton, Shyamala Dakshinamurti, Ruth Graham, Christopher D. Pascoe, Amir Ravandi, Biniam Kidane

**Affiliations:** 1 Biology of Breathing Research Theme, the Children’s Hospital Research Institute of Manitoba, Winnipeg, MB R3E 3P4, Canada; 2 Section of Thoracic Surgery, Department of Surgery, Rady Faculty of Health Sciences, Max Rady College of Medicine, University of Manitoba, Winnipeg, MB R3A 1R9, Canada; 3 Undergraduate Medical Education (UGME), Max Rady College of Medicine, University of Manitoba, Winnipeg, MB R3E 3P5, Canada; 4 Department of Physiology and Pathophysiology, Rady Faculty of Health Sciences, Max Rady College of Medicine, University of Manitoba, Winnipeg, MB R3E 0J9, Canada; 5 Department of Anesthesiology, Perioperative and Pain Medicine, Max Rady College of Medicine, University of Manitoba, Winnipeg, MB R3E 0Z2, Canada; 6 Institute of Cardiovascular Sciences, St. Boniface Hospital Research, University of Manitoba, Winnipeg, MB R2H 2A6, Canada; 7 Department of Biomedical Engineering, Price Faculty of Engineering, University of Manitoba, Winnipeg, MB R3T 5V6, Canada

**Keywords:** plasma, lipidomics, porcine, one-lung-ventilation, acute lung injury, postoperative complications, inflammation

## Abstract

One-lung ventilation (OLV) is performed during lung surgeries by ventilating a single lung, while collapsing the operative lung to provide surgical exposure within the thoracic cavity. While a lung-protective ventilation strategy is recommended during OLV, increasing the fraction of inspired oxygen (FiO_2_) or tidal volume (V_T_) may be required to prevent hypoxemia during surgery. Unfortunately, these increases are associated with postoperative lung injury. Using a porcine model of OLV, our project aims to determine if high FiO_2_ or V_T_ during OLV contributes to elevation of pro-inflammatory lipid mediators postoperatively. Fifteen three-month-old farm-bred pigs underwent left upper lobectomy requiring OLV. Pigs were exposed to one of three ventilation parameters: normoxic low V_T_ lung-protective ventilation LPV-NO, *n* = 5, FiO_2_ < 50%, V_T_ = 6 mL/kg), hyperoxic lung-protective ventilation (LPV-HO, *n* = 5, FiO_2_ >100%, V_T_ = 6 mL/kg), or normoxic high V_T_ (injurious mechanical ventilation) (IMV, *n* = 5, FiO_2_ < 50%, V_T_ = 10–12 mL/kg). Arterial plasma was collected before and after OLV, and lipids were detected via LC-MS-MS. Lipidomic analysis demonstrated a statistically significant increase (FC = 2, *p* ≤ 0.05) in lysophosphatidylethanolamines (LPE 18:3, LPE 20:4, LPE 18:2, LPE 22:6), free fatty acids (FFA 20:4), phosphatidylserine (PS 38:5), lysophosphatidylcholine (LPC 18:1, LPC 18:3, LPC 22:6), triglyceride (TG 18:2-18:2-20:4), free fatty acids (FFA 20:5), linoleyl-carnitine molecules (C18-2 Linoleoyl Carnitine), and phosphatidylethanolamines (PE 36:5) in LPV-HO. IMV resulted in a significant increase (FC = 2, *p* ≤ 0.05) in triglyceride (TG 18:2-18:2-20:4), diglyceride (DG 18:1-20:4, DG 16:0-20:4), linoleyl-carnitine molecules (C18-2 Linoleoyl Carnitine), and free fatty acids (FFA 20:5). There was no significant change in lipid biomarker levels following LPV-NO post-surgery. Our lipidomic analysis supports that both high FiO_2_ and V_T_ contribute to systemic lipid metabolic derangements. Lipids that were elevated in LPV-HO and IMV are associated with multiple inflammatory pathways implicated in lung injury. This suggests that intra-operative anti-inflammatory therapies targeted to these lipid pathways may reduce or prevent postoperative pulmonary complications after lung surgery.

## 1. Introduction

Lung cancer is the leading cause of cancer death worldwide, with high mortality and morbidity [1]. In 2026, it is estimated that there will be over 32,000 new lung cancer diagnoses and over 19,000 deaths due to lung cancer in Canada alone [2]. In early-stage disease, lung resection surgery is a definitive cure for lung cancer. However, lung surgeries have a high rate of postoperative respiratory complications, with one of the most severe complications being acute respiratory distress syndrome (ARDS) [3]. ARDS is characterized by acute inflammation that disrupts the lung endothelial and epithelial barriers, leading to loss of the alveolar-capillary membrane integrity, neutrophil infiltration, and release of pro-inflammatory molecules [4]. Clinically, this presents as hypoxemia, diffuse radiographic opacities, increased alveolar dead space, and decreased lung compliance [4]. The incidence of ARDS after lung resection is estimated between 2.45 and 4.3%, with mortality rates of 24.6–40% [5,6,7,8].

Main contributors to the development of ARDS postoperatively include ventilator-induced lung injury (VILI) and surgical trauma. Lung-protective ventilation strategies are employed to prevent the occurrence of ARDS due to VILI, and this has led to a decrease in the incidence of ARDS [9,10,11,12]. These ventilation strategies include maintaining tidal volume (V_T_) at 6–8 mg/kg and plateau pressure below 30 cm H_2_O while keeping fraction of inspired oxygen (FiO_2_) as low as possible [9]. Mechanical ventilation during lung resection surgery is especially high risk for the development of VILI, as one-lung ventilation (OLV) is required [13]. OLV involves collapsing the operated lung, while all ventilatory stress is placed on the contralateral lung. OLV enables the usage of minimally invasive surgical techniques, improving the safety and outcomes of patients following lung surgery [14]. During OLV, lung-protective ventilation strategies are also employed to prevent lung injury [15]. However, a challenge of OLV is the management of hypoxia and hypercapnia throughout surgery; this requires modifying ventilation settings by increasing the FiO_2_ or V_T_, amongst other strategies [16]. However, increases in FiO_2_ and V_T_ are associated with postoperative pulmonary complications, including ARDS [10,17,18,19].

High V_T_ causes injury via volutrauma due to overdistension [18], whereas high FiO_2_ increases the presence of reactive oxygen species (ROS), leading to lung damage [20]. In the presence of ROS, membrane phospholipids undergo peroxidation, and high levels of lipid oxidation can initiate inflammatory cascades in cells [21]. Moreover, excessive ROS leads to disruption of the plasma membrane and propagation of ROS production, amplifying tissue injury [22,23]. Thus, lipid peroxidation may be a contributing factor to VILI in OLV and the development of ARDS postoperatively.

Our lab previously developed a porcine model of OLV to adequately assess the factors contributing to VILI in OLV [24]. We hypothesized that high FiO_2_ or high V_T_ during OLV may cause systemic increases in pro-inflammatory lipid mediators postoperatively. To address this, pigs underwent a left upper lobectomy while being exposed to either lung-protective ventilation, hyperoxia, or high V_T_. Blood samples were taken before and after surgery and analyzed for changes in lipid biomarkers.

## 2. Results

**Figure 1:**
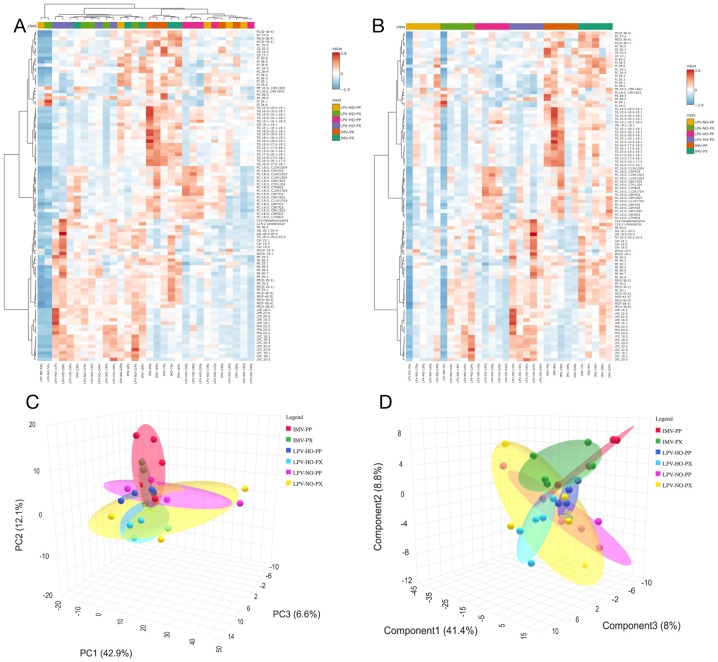
Clustered (**A**) and unclustered (**B**) heat map displaying lipid abundance in top 100 expressed lipids in blood plasma of pigs before and after undergoing left upper lobectomy with either LPV-NO, LPV-HO, or IMV. 3D model representation showing significant overlap amongst confidence ellipses of the groups, but no clear separation between ventilation groups and time points in both (**C**) PCA and (**D**) PLSDA analysis of the overall lipid characteristics of blood plasma in pigs either before or after left upper lobectomy with LPV-NO, LPV-HO, or IMV. Group legend: LPV-NO-PP = lung-protective ventilation with normoxia pre-OLV. LPV-NO-PX = lung-protective ventilation with normoxia post-OLV. LPV-HO-PP = lung-protective ventilation with hyperoxia pre-OLV. LPV-HO-PX = lung-protective ventilation with hyperoxia post-OLV. IMV-PP = injurious mechanical ventilation pre-OLV. IMV-PX = injurious mechanical ventilation post-OLV.

In the present study, we wanted to quantify the changes to the systemic lipidome following exposure to lung-protective ventilation with normoxia (LPV-NO), lung-protective ventilation with hyperoxia (LPV-HO), or injurious high V_T_ mechanical ventilation (IMV) during lung surgery. We identified 26 distinct lipid classes (Figure S1) and performed an initial analysis of the total lipid class alone. The lipid abundance features observed in the heatmap, Partial Least Squares Discriminant Analysis (PLS-DA), and VIP Scores identified the predominant lipid classes driving the greatest changes across groups as PC, OxPC, PI, PC(O), PE, DG, PE(P), and carnitine. Interestingly, however, there were no significant changes in either the LPV-NO or the IMV groups following surgery; only the LPV-HO group showed a significant increase in Total LPE (Figure S1D–I). We then proceeded to perform a more integrated analysis, including all lipid subclasses, totalling 340 lipids. Clustered lipid abundance shows increased relative abundance in the IMV-pre and IMV-post groups when compared to LPV-NO and LPV-HO groups (Figure 1A,B). Interestingly, the LPV-HO post shows increased relative abundance in comparison to LPV-HO pre group. Lastly, the LPV-NO pre and LPV-NO post have similar levels of lipid abundance across samples (Figure 1A,B). Principal Component Analysis (PCA) of all groups was performed to determine the amount of similarity between conditions when considering all lipids detected (Figure 1C). PCA showed significant overlap amongst confidence ellipses of the groups and did not show clear separation between ventilation groups and time points. Component 1 of PCA explained 44.3% of the variance, whereas component 2 explained 12.2% of the total variance. Partial Least Squares Discriminant Analysis (PLSDA) of all groups had similar results, as there was significant overlap between group confidence ellipses and no clear separation between them (Figure 1D). PLSDA component 1 explained 42.8% of the variance, whereas component 2 explained 8.6% of the variance in the groups.

**Figure 2:**
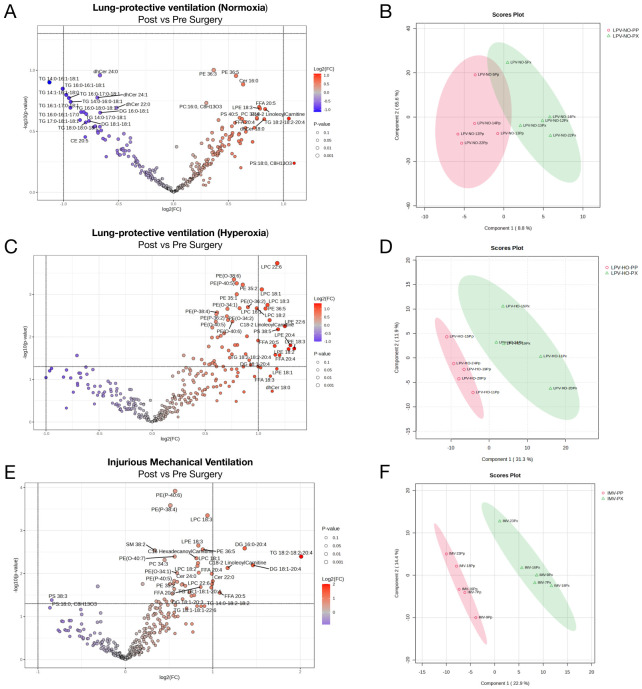
Volcano plots displaying the change in abundance of the top 100 expressed lipids following left upper lobectomy with LPV-NO (**A**), LPV-HO (**C**), or IMV (**E**) groups. PLSDA plots comparing overall lipid characteristics of blood plasma in pigs before and after left upper lobectomy with LPV-NO (**B**), LPV-HO (**D**), or IMV (**F**) mechanical ventilation. Group legend: LPV-NO-PP = lung-protective ventilation with normoxia pre-OLV. LPV-NO-PX = lung-protective ventilation with normoxia post-OLV. LPV-HO-PP = lung-protective ventilation with hyperoxia pre-OLV. LPV-HO-PX = lung-protective ventilation with hyperoxia post-OLV. IMV-PP = injurious mechanical ventilation pre-OLV. IMV-PX = injurious mechanical ventilation post-OLV.

To obtain information on the lipids that are altered following OLV, volcano plots were employed to visualize the change in lipids following OLV compared to before surgery (Figure 2A,C,E). The LPV-NO group did not show any significant changes in lipids following OLV. This is consistent with PLSDA analysis of the LPV-NO pre vs. LPV-NO post condition, where there was no clear distinction between lipid profiles (Figure 2B). The LPV-HO condition had a significant increase in the abundance of 13 lipids following OLV (FC = 2.0, *p* < 0.05), namely, lysophosphatidylethanolamine (LPE) 18:3, LPE 20:4, LPE 18:2, LPE 22:6, free fatty acid (FFA) 20:4 (arachidonic acid), phosphatyidyl serine (PS) 38:5, lysophosphatidylcholine (LPC) 22:6, triglyceride (TG) 18:2-18:2-20:4, FFA 20:5, C18-2 Linoleoyl Carnitine, LPC 18:3, phosphatidylethanolamine (PE) 36:5, and LPC 18:1. The IMV condition had significant increases in the abundance of 5 lipids (FC = 2.0, *p* < 0.05): TG 18:2-18:2-20:4, diglyceride (DG) 18:1-20:4, DG 16:0-20:4, C18-2 Linoleoyl Carnitine, FFA 20:5. PLS-DA plots of the LPV-HO and IMV conditions both show clear distinction between the post vs. pre-OLV groups, respectively (Figure 2D,F). The group variations observed in Components 1 and 2 in both Figure 2D and Figure 2F correlate with the observed significant lipids upregulated in Figure 2C,E, indicating which lipids may be driving the percentage changes in PC components in PLSDA results. Table 1 and Table 2 are displayed showing the name, fold change (log2), and raw *p* value significance (−log10) of all increased lipids within the LPV-HO and IMV conditions. There were no lipids that demonstrated a significant decrease in abundance following OLV in any of the three conditions.

**Figure 3:**
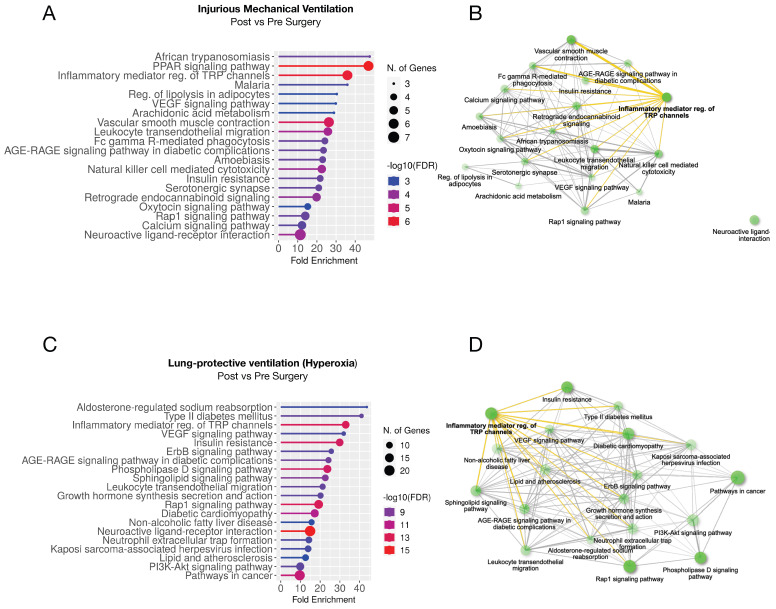
To further understand the interactions of the unregulated lipids from volcano plots, pathway enrichment analysis was performed using the STRING database and the Kyoto encyclopedia of genes and genomes (KEGG) and then visualized using ShinyGO 0.80 (high interaction confidence: >0.7). KEGG enrichment analysis of top 20 pathways altered following OLV with either injurious mechanical ventilation/IMV (**A**) or lung-protective ventilation/LPV-HO group (**C**) and corresponding STRING analysis (**B**,**D**) based on significantly altered plasma lipids.

**Figure 4:**
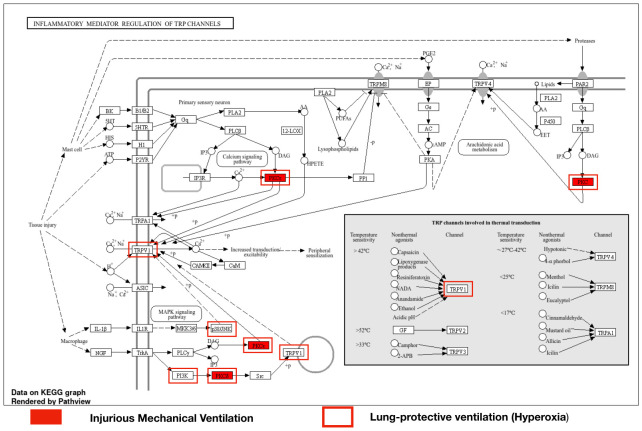
Pathway visualization of the inflammatory mediator regulation of TRP channel pathway. The predicted interactions between pathway proteins and lipids upregulated in LPV-HO (designated by a red border around proteins) or IMV (proteins highlighted in red) are highlighted.

Figure 3A,C show the top 20 most-enriched pathways altered in LPV-HO and IMV. To generate this pathway analysis, the significant lipids identified in both LPV-HO and IMV groups were first used to predict the top 15 proteins that each lipid may interact with, which then allowed for the enrichment analysis performed. Here, the Lollipop plots display the fold enrichment on the *x*-axis and the number of interacting genes in each pathway based on circle size. Colour of the lollipop plot corresponds to the −log10 of the false discovery rate of each pathway. Interaction networks corresponding to lollipop plots for IMV and LPV-HO conditions are shown in Figure 3B,D. Pathways that were enriched in both conditions include the inflammatory mediator regulation of transient receptor potential (TRP) channel pathway, VEGF signalling pathway, Insulin resistance pathway, ACE-RAGE pathway in diabetic complications, leukocyte trans-endothelial migration pathway, and Rap1 signalling pathway. As the inflammatory mediator regulation of TRP channel pathway was one of the top three enriched pathways in both the IMV and LPV-HO groups, we used Pathview Web to visualize the cell signalling involved (Figure 4). Proteins that interact with the upregulated lipids are highlighted in red. The primary protein interactions in this pathway involve the protein kinase C (PKC) family of proteins in both our experimental conditions. LPV-HO was also found to interact with phosphoinositide 3-kinase (P13K), p38/c-Jun N-terminal Kinase (JNK), and transient receptor potential vanilloid 1 (TRPV1) receptors.

**Figure 5:**
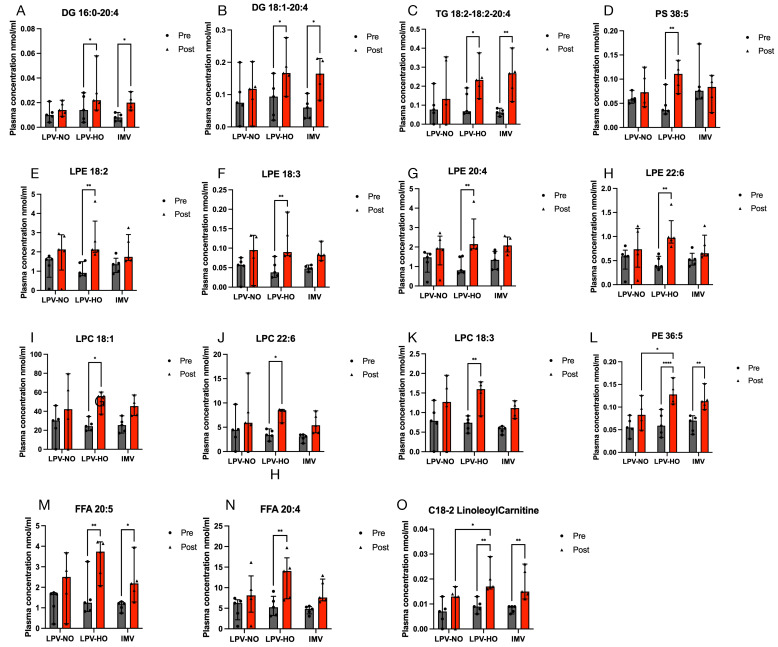
Plasma lipids in pigs were measured following left-upper lobectomy with one of three OLV mechanical ventilation parameters: LPV-NO, LPV-HO, or IMV. (**A**–**O**) Plasma lipid concentrations significantly increased in either the LPV-HO condition, IMV condition, or both, but no significant change in any plasma lipids following LPV-NO. All lipids that had a statistically significant change in abundance following OLV are displayed. Analysis performed by two-way ANOVA with Tukey’s post hoc test (*, *p* < 0.05, **, *p* < 0.01, ****, *p* < 0.0001). LPV-NO = lung-protective ventilation—normoxia, LPV-HO = lung-protective ventilation—hyperoxia, IMV = injurious mechanical ventilation, DG = diglyceride, TG = triglyceride, PS = phosphatidylserine, LPE = lysophosphatidylethanolamine, LPC = lysophosphatidylcholine, PE = phosphatidylethanolamine, FFA = free fatty acid.

Figure 5 shows the change in plasma concentration for all lipids that were found to be significantly increased in either the LPV-HO condition, IMV condition, or both. There was no significant change in any plasma lipids following LPV-NO. Interestingly, six lipids had significantly increased concentrations following OLV in both LPV-HO and IMV. These lipids include FFA 20:5, PE 36:5, C18-2 Linoleoyl Carnitine, DG 18:1-20:4, DG 16:0-20:4, and TG 18:2-18:2-20:4. There were also seven lipids that were increased in the LPV-HO condition only following OLV, namely, FFA 20:4, PS 38:5, LPE 18:2, LPE 18:3, LPE 20:4, LPE 22:6, LPC 18:1, LPC 18:3, and LPC 22:6.

## 3. Discussion

Our current study is the first to our knowledge to demonstrate that systemic lipid derangements are occurring following harmful hyperoxic ventilation and high V_T_ ventilation during OLV. Using LC/MS/MS, we were able to identify multiple lipid molecules that were elevated following harmful mechanical ventilation during lung surgery. Enrichment analysis of the elevated lipids shows potential pathways that may be contributing to VILI following lung surgery. These pathways can present as future targets for studies to further understand the molecular underpinnings of lung injury and ARDS due to OLV.

Observing the heatmap analysis found in Figure 1A,B, there appears to be variability between animals, as one animal within the LPV-NO group (LPV-NO 5) had a much different lipid abundance pattern than all other animals. This is further supported by the clustered heatmap (Figure 1A), where the pre- and post-OLV conditions from LPV-NO 5 form a cluster by themselves. Within the heatmaps, we also see that the IMV group had a higher general abundance of lipids prior to OLV compared to the other conditions. This is unexpected, as ventilation parameters were the same up until sample collection. Despite this potential variation, our PCA and PLSDA analysis (Figure 1C,D) indicate that biologic groups are all behaving similarly, as we see substantial overlap between groups.

Examining the volcano plots in Figure 2, there is a shift to the right for the LVP-HO and IMV groups but not the LPV-NO group. This suggests that there is an increase in plasma lipids following LPV-HO and IMV, but relative lipid content remains the same following LPV-NO. We believe that this may be due to spilling of surfactant lipids and alveolar cell membrane lipids into systemic circulation due to damage caused by harmful ventilation parameters. Surfactant is produced by alveolar type II cells and is composed of 90% lipids and 10% proteins [25]. The composition of lipids in surfactant primarily consists of phospholipids such as phosphatidylcholines (PC), phosphatidyl glycerol, PE, and PS [25,26,27]. Our results show an increase in serum of PE, PS, and various LPE and LPC species in plasma, all of which are unsaturated or polyunsaturated fatty acids. Previous studies have demonstrated that patients with ARDS have decreased lipid abundance compared to healthy controls, increased fractional concentrations of unsaturated and polyunsaturated fatty acids (UFAs and PUFAs) in BALF, and that these lipids are characteristic of cell membrane materials [27,28,29]. Furthermore, circulating surfactant protein D, a primary protein found in lung surfactant, has been proposed as a potential biomarker for ARDS [30]. Indeed, V_T_ of 10 mL/kg has been previously shown to cause diffuse alveolar damage in pigs during OLV, predominantly in the ventilated lung [31]. High FiO_2_ leads to increased ROS, resulting in oxidation of cellular lipids and proteins, mitochondrial dysfunction, DNA damage, loss of membrane integrity and cell death [20,32,33]. Together, this supports that the relative increase in lipids following LPV-HO and IMV in blood may be due to local lung damage spilling into systemic circulation; however, more work would be required to confirm this. It is possible that changes in lipid concentration can be due to dietary intake of lipids; however, this would be unlikely in this case, as high levels of TGs are expected following dietary intake of fats, and we only saw a significant increase in 1 of the 44 TGs detected within our analysis (Figure 2 and Figure 5). Figure 5 displays all lipids that were significantly elevated following OLV, and their corresponding concentration in blood plasma. Multiple lipids were found to be significantly elevated, but did not have an FC > 2.0, so they were excluded from the volcano plots in Figure 2.

Within the LPV-HO group, there was a substantial increase in multiple LPC and LPE species (Figure 5). LPCs and LPEs are primarily produced as metabolites of enzymatic cleavage of PC and PE via phospholipase A2 (PLA2), with arachidonic acid (AA) also being liberated in the process [34]. PLA2s have also been implicated in lung injury and ARDS [34,35]. Secreted PLA2 (sPLA2) is the largest subfamily of PLA2, containing 11 isoforms, and is secreted in the lungs primarily by alveolar macrophages and mast cells during inflammation [36]. sPLA2 catabolizes surfactant phospholipids, damaging and depleting the surfactant, and its expression is increased in hyperoxic states due to increased ROS [37,38]. In ARDS, dipalmitoyl phosphatidylcholine (DPPC), the main surfactant phospholipid, is hydrolyzed, and this leads to an accumulation of lyso-PCs [39], and sPLA2 isoforms have been shown to hydrolyze DPPC [37]. LPCs have also been shown to cause acute endothelial cell disruption in pulmonary endothelial cell culture, which is a contributing factor to the development of ARDS [40]. Increased levels of LPC and LPE in plasma support that lung injury is occurring during LPV-HO, and elevated levels of LPC and LPE may also be linked to increased expression of sPLA2s and increased lipid catabolism in our model, but that is yet to be elucidated.

We also observed increased levels of FFA 20:5 (Eicosapentanoic acid, EPA) in both IMV and LPV-HO, and increased levels of FFA 20:4 (arachidonic acid, AA) in LPV-HO (Figure 5). AA is produced by the hydrolysis of phospholipids by PLA2 and can be further metabolized into eicosanoids. This can be accomplished by cyclo-oxygenases to produce prostaglandins and thromboxanes, lipoxygenases to produce leukotrienes, lipoxins and hydoryeicosatetrenoic acids (HETEs), or cytochrome P-450 to produce epoxygenases [41]. AA and its metabolites have been implicated in both propagation and resolution of inflammation, depending on the pathway involved [41,42,43,44,45,46]. EPA, on the other hand, has primarily been recognized for its anti-inflammatory properties but also has pro-inflammatory potential [47,48]. Thus, we cannot conclude if the elevation of AA and EPA is due to propogation of pro-inflammatory or anti-inflammatory physiological responses, although, based on the ventilation parameters being utilized in this study and the temporal relationship of the sampling to the insult, it is more likely that these are pro-inflammatory processes rather than anti-inflammatory responses.

Using bioinformatic techniques and databases, we were able to complete an enrichment analysis of molecules that have a high likelihood of interacting with upregulated lipids found in Table 1 and Table 2. Both the IMV and LPV-HO conditions had significantly enriched pathways that are associated with lung injury and ARDS (Figure 3). For instance, both conditions showed enrichment of the inflammatory mediator regulation of TRP channel pathway, leukocyte trans-endothelial migration pathway, and Rap1 signalling pathway (Figure 3A,C). TRP channels are ion channels found on immune cells that, when stimulated, induce an increase in pro-inflammatory cytokines, including IL-6, IL-1beta, and TNF-alpha [49]. Notably, oxylipins from FFA [50] and LPCs [51] can activate TRP channels, namely, TRPV4. Neutrophils play a key role in endothelial injury described in ARDS, and multiple subtypes of TRPs have been described in neutrophils. Activation of TRPs on neutrophils leads to enhanced production of inflammatory mediators [49]. Rap1 signalling has been shown to promote barrier function and decrease permeability of lung vascular endothelium, whereas the leukocyte trans-endothelial migration is involved in neutrophil infiltration of the lungs, a classical hallmark of ARDS [52,53,54,55].

As the inflammatory mediator regulation of TRP channel pathway was one of the top three enriched pathways within both IMV and LPV-HO, we used pathway to visualize the proteins that are most likely to interact with upregulated lipids (Figure 4). Lipids in both the IMV and LPV-HO conditions both appear to primarily interact with the protein kinase C (PKC) family of proteins within this pathway, including PKC-delta and PKC-epsilon. LPV-HO also appears to have interactions with P13K, p38/JNK, and TRPV1 proteins; however, this was not found in IMV.

PKCs have been shown to contribute to the pathogenesis of ARDS, and inhibition of PKCs results in decreased inflammatory response and lung injury in murine models [56,57]. PKC-delta knockout in mice has also been shown to decrease endothelial barrier dysfunction in lung injury [58]. The p38/JNK pathway is activated in hyperoxic cell death, and inhibition of p38/JNK improves cell survival in cell culture [59]. However, JNK-deficient mice had increased susceptibility and lung epithelial cell apoptosis, suggesting that this pathway is protective transiently but may be harmful with long-term hyperoxia exposure [60,61]. TRPV1 channels are modulators of tissue inflammation in bronchial and alveolar epithelial cells; inhibition of TRPV1 decreases inflammation and oxidative stress in a COPD cell culture model [62]. To our knowledge, there were no studies exploring the role of TRPV1 in models of ARDS. Interestingly, the TRPV4 isoform has been more extensively explored in its role in lung injury and ARDS, and it has been demonstrated to interact with PKC [63,64,65]. Together, these enrichment analyses provide multiple future targets to be considered to further understand the pathways and proteins that are being activated in our OLV model.

There are limitations to our study, as it is not known if the lipid composition of plasma found in pigs is similar to that of humans. Secondly, metabolomic and proteomic data of pigs are not as robust as those of other animal models, such as mice or rats. This presented as a challenge when we completed enrichment analysis, and as such, we were required to use predicted human protein interactions for the elevated lipids discovered in our pig model. This switching of species within our analysis presents a potential bias, whereby there may be a difference in lipid alterations in humans compared to pigs, thus leading to alternate pathways being enriched compared to what our study found. Therefore, cross-species inference (predicting human protein interactions from porcine lipid data) could skew enrichment results for specific pathways [66]. While some major metabolic pathways, such as mTOR and FoxO, among others, may be evolutionarily conserved across various species in protein bonding sites [67], some lipid–protein interactions may not necessarily be conserved between both pigs and humans [68,69]. Even so, recent studies still suggest that pigs are a scientifically important intermediate species (rodent–human) for scientific research on human health [70]. Despite this challenge regarding lipidomics, using an animal model allows us to use healthy subjects and minimize confounding variables that are difficult to control for in human OLV trials [12,71]. Moreover, pig models have previously been used as a model for ALI studies and act as ideal and preferred large animal models to perform lung surgery on, as lung anatomy is relatively similar to that of human lung anatomy in size and structure [72,73].

We also acknowledge that the sample size is small and cannot be used to generate definitive conclusions and should be considered hypothesis-generating. Given that these are not transgenic animals, there will be heterogeneity in the pigs, and thus it is possible that some of the differences identified in both clinical, physiological and molecular responses to the experiment may be heavily influenced by this baseline heterogeneity. To mitigate this, we chose 3-month-old young pigs so as to limit the variability in the pulmonary (and other) exposures experienced by the pigs prior to surgery and OLV. This increases the homogeneity of the pig lungs and thus lessens the confounding when trying to interpret the tissue damage that we observe after surgery and OLV. Another limitation is that we were only able to analyze plasma lipids in our experiments. Plasma lipid contents reflect the systemic immune response to harmful mechanical ventilation but give limited insight into the local response to ventilation occurring in the lungs. Although we could not determine the local response in this study, future directions of this research involve examining the lipidomic derangements in bronchoalveolar lavage fluid (BALF) and quantifying biological changes that occurred in the lung tissue to address this. Moreover, blood samples were collected only preoperatively and at a single postoperative time point; hence, the dynamic trajectory of lipid changes remains unexplored. This is currently a limitation, and future studies may need to incorporate multiple time points (e.g., intra-operative intervals, 6 h and 24 h postop) to capture the temporal evolution of the lipidome. Despite this, there are also benefits to examining plasma derangements, as retrieving peripheral blood samples is easily accessible and less invasive compared to attempting to collect a local sample, such as BALF, to assess for inflammation. Moreover, blood sampling of disease biomarkers is already used extensively clinically. Thus, it is more desirable to examine blood plasma for markers of lung injury clinically, as it would be quicker and easier to obtain, and less invasive for patients.

A last limitation of our study is that there is currently no biological data to supplement our enrichment analysis that shows increased interactions with multiple pro-inflammatory pathways, such as the “inflammatory mediator regulation of TRP channel pathway”. Therefore, the discussion surrounding the impact of the elevated lipids found in our study on these pathways is based on speculation and similar findings from other studies that are not within our model. In the future, we plan to pair this computational data with biological assays to determine if the lipids we identified influence the protein pathways that were highlighted.

Interestingly, we did not see a change in levels of oxidized phospholipids (Ox-PLs) such as ox-PCs (oxidized phosphatidylcholine), ox-PS (oxidized phosphatidylserine), or ox-PE (oxidized phosphatidylethanolamine) in plasma, which was unexpected. This might be due to the pigs’ relative health and intact antioxidant systems. However, the result could be different if using a disease model. Ox-PLs are known to be important pro-inflammatory mediators and regulators of immune response in lung injury [74,75]. To our knowledge, there is limited literature supporting the absence of OxPLs in pig models of OLV, which contributes to the contradictory general understanding of OLV-induced oxidative stress. Although we did not see changes in oxidized phospholipid concentrations systemically, this does not preclude the presence of these molecules within the local inflammatory environment. It is possible that the local inflammatory response was not strong enough or did not last for a long enough time to generate a substantial change in these molecules systemically. Alternatively, there could have been a lack of local OxPL production and signalling within our model. OxPLs are increased in states of hyperoxia and lung injury, and future work is needed to determine if there is an increase in the abundance of OxPLs within the lungs following harmful OLV in our model.

## 4. Materials and Methods

Porcine model: This study was approved by the Bannatyne Campus Animal Care Committee (Protocol Reference Number: 21-013 (AC11692)). Fifteen three-month-old farm-bred pigs were procured from an approved farm by the University of Manitoba Animal Services and were randomly assigned to either the control group with lung-protective ventilation with normoxia (LPV-NO) (*n* = 5, V_T_ = 6 mL/kg, PIP < 30 cm H_2_O, positive end-expiratory pressure (PEEP) = 5 cm H_2_O, I:E ratio = 1:2, FiO_2_ ≤ 50%), lung-protective ventilation with hyperoxia (LPV-HO) (*n* = 5, V_T_ = 6 mL/kg, PIP ≥ 30 cm H_2_O, PEEP = 5 cm H_2_O, I:E ratio = 1:2, FiO_2_ = 100%), or the injurious mechanical ventilation (IMV) (*n* = 5, V_T_ = 8–10 mL/kg, PIP < 30 cm H_2_O, PEEP = 5 cm H_2_O, I:E ratio = 1:2, FiO_2_ ≤ 50%) group [24]. The sex of the pigs was randomized. All pigs underwent left upper lobectomy. Pigs were sedated with intramuscular xylazine (1 mg/kg) (Bimeda, Cambridge, ON, Canada), ketamine (10 mg/kg) (Baxter, Mississauga, ON, Canada), and atropine (0.01 mg/kg) (Sandoz, Boucherville, Quebec, ON, Canada). Induction of anesthesia was completed with inhaled isoflurane (4%) (Baxter, Mississauga, ON, Canada). A 7.0 single-lumen cuffed endotracheal tube (ETT) was placed using direct laryngoscopy and local anesthetic to prevent laryngospasm. After intubation, total intravenous anesthesia (TIVA) was initiated using an infusion of propofol (Baxter, Mississauga, ON, Canada), ketamine, and rocuronium (10/2.5/1 mg/kg/hour) (Pfizer, Kirkland, QC, Canada) to permit the use of an intensive care ventilator (Esprit^®^, Murrysville, PA, USA). Prior to OLV, both lungs were ventilated to establish normal airflow before surgery. Next, two lung ventilation (TLV) was maintained with V_T_ of 10 ± 2 mL/kg, minute ventilation (Ve) of 7 ± 1 L/min, an inspiratory/expiratory (I:E) ratio of 1:2, peak inspiratory pressure (PIP) 20 ± 5 cm H_2_O, mean airway pressure (MAP) 8.5 ± 0.5 cm H_2_O, PEEP of 5 ± 0.5 cmH_2_O, a FiO_2_ of 0.30 ± 0.5, and the respiratory rate adjusted to maintain normal arterial partial pressure of CO_2_ (PaCO_2_) (i.e., 40 ± 5 mmHg). Animals were allowed to stabilize on TLV for 15 min. Following this, arterial blood samples were acquired. While in the supine position, a bronchial blocker (EZ Blocker, Wayne, PA, USA) was placed and inflated in the left bronchi using bronchoscopy, initiating one-lung ventilation. Animals were then allowed to stabilize on OLV for 15 min. Pigs were then placed in the right lateral decubitus position and allowed to stabilize on OLV for 15 min. Following this, a left lateral thoracotomy was performed. Intercostal nerve block using bupivacaine (0.25%) (Pfizer, Kirkland, QC, Canada) was administered, and anatomical resection of the left upper lobe was completed. Pigs were then allowed to stabilize on OLV for 15 more minutes. Following closure of the incision, the endotracheal blocker was removed, and TLV was reestablished, and the animal was allowed to stabilize on TLV for 15 min. Blood samples were taken after 15 min of TLV. The experiment was terminated by performing a bilateral clamshell thoracotomy and injecting 100 mEq/kg KCl (Baxter, Mississauga, ON, Canada) directly into the heart. The inferior vena cava was then ligated, and ventilation was terminated. The lungs were then removed en bloc and stored in Krebs–Henseleit buffer (Thermo Fisher Scientific, Waltham, MA, USA) on ice for postoperative analysis.

Lipid Extraction: Lipid extraction and separation have been previously described [76]. Lipid extraction of arterial plasma samples was done using chloroform (Sigma-Aldrich, Oakville, ON, Canada) and methanol (Thermo Fisher Scientific, Mississauga, ON, Canada). A total of 10 μL of plasma was added to a 1.5 mL Eppendorf tube (Fisher Scientific, Mississauga, ON, Canada). A total of 30 μL of lipid internal standards (ISTD) in chloroform/methanol (1:1, *v*/*v*) and 200 μL chloroform/methanol (2:1, *v*/*v*) was added to each tube. A complete list of ISTDs used is provided in Table S1. The mixture was vortexed for 10 min on a rotary mixer, then sonicated in a room-temperature water bath for 30 min. The mixture was rested for 20 min at room temperature, then centrifuged for 20 min at room temperature (RT) (20,000× *g*). The lipid phase was transferred to a clean Eppendorf tube and dried using nitrogen gas at room temperature. Lipids were then reconstituted in 50 μL of water-saturated 1-butanol (Sigma-Aldrich, Oakville, ON, Canada) and sonicated in a water bath for 10 min at room temperature. Then, 50 μL of 10 mM ammonium formate (Sigma-Aldrich, Oakville, ON, Canada) in methanol was added to the lipid extract and centrifuged (10,000× *g*, 10 min, RT). A total of 80 μL of supernatant was transferred into the micro insert in sample vials for lipid analysis.

Lipid Separation: Lipids were separated using reverse-phase liquid chromatography-electrospray ionization tandem mass spectrometry (LC/ESI-MS/MS) using a prominence chromatographic system. Analyst v1.6 and Multi-Quant v2.1 software were used for instrument control and data processing. Separation was performed on a Zorbax C18, 1.8 um, 50 × 2.1 mm column (Agilent Technologies, Mississauga, ON, Canada) with a flow rate of 300 μL/min using a linear gradient of mobile phase A and mobile phase B. Mobile phase A consisted of tetrahydrofuran (Sigma-Aldrich, Oakville, ON, Canada), methanol, and water (20:20:60, *v*/*v*/*v*) and 10 mM ammonium formate. Mobile phase B contained tetrahydrofuran, methanol, and water (75:20:5, *v*/*v*/*v*) and 10 mM ammonium formate. The following elution protocol was implemented: start with 0% of solvent B; increase to 100% B at 8 min; maintain at 100% B for 2.5 min; back to 0% B over 0.5 min. The column was re-equilibrated for 3 min to the original conditions (0% solvent B) prior to the next sample injection. A total of 5 μL of sample was injected for each run. Throughout the duration of the analysis, the analytical column was maintained at 50 °C, and the autosampler was maintained at 25 °C.

Data Analysis: To accomplish this, we collected arterial blood plasma samples prior to the initiation of OLV and after termination of OLV. Plasma samples subsequently underwent lipidomic analysis. A total of 340 unique lipids were detected in the pig blood plasma, stemming from 26 distinct lipid classes (total lipids-only analysis available in Supplemental Figure S1). Lipid data were uploaded into MetaboAnalyst 5.0, a web-based platform for comprehensive metabolomics data analysis. Hierarchical clustering was visualized using heatmaps to display lipid abundance. Data were analyzed using an unsupervised PCA analysis, a supervised PLSDA and volcano plot analysis of statistical significance (*p* < 0.05) versus magnitude of change (fold change, FC > 2.0) to identify altered lipids. All data analysis was completed using Metaboanalyst v5.0. Bar graphs were analyzed in GraphPad (Prism 10) using two-way ANOVA with Tukey’s post hoc *t*-test (*p* < 0.05). While there was an observed divergent lipid profile in pig #5 when compared to others in the same LPV-NO group, no particular underlying reasons (e.g., individual health status, dietary differences) were identified that could justify it. However, as previously mentioned in the Discussion section, the sample size (*n* = 5) is small and cannot support definitive conclusions. Given that these are not transgenic and that pigs are heterogeneous, it is possible that some of the differences identified in clinical, physical, and molecular responses to the experiment may be heavily influenced by this baseline heterogeneity, which we tried to mitigate by choosing 3-month-old young pigs to limit the variability in the pulmonary (and other) exposures experienced by the pigs prior to surgery and OLV. An outlier test was not performed; however, we performed the same lipid analysis at the same level by including and excluding pig 5, and no significant differences were observed in the outcome results between the two; hence, we proceeded with all subsequent analyses of all pigs, including pig 5. All lipids that had a statistically significant change were searched within the Human Metabolome Database (HMDB) to retrieve each molecule’s unique Simplified Molecular Input Line Entry System (SMILES) code. Each lipid was then input into Swiss Target Prediction to identify interacting proteins with each lipid. Once all identifying proteins were identified, the STRING database was used for enrichment analysis (high interaction confidence > 0.7) using the Kyoto Encyclopedia of Genes and Genomics (KEGG) to determine which molecules and pathways are the most probable targets of the altered lipids. Data were input into ShinyGO 0.80 (URL accessed on 19 September 2024: https://bioinformatics.sdstate.edu/go/) for visualization.

## 5. Conclusions

Collectively, our results indicate that lipid inflammatory processes associated with lung injury are occurring intraoperatively when exposed to harmful ventilation. This has broad implications for the management of patients on OLV, as exposing patients to extended periods of hyperoxia or increased V_T_ can trigger activation of inflammation, increasing the likelihood of postoperative respiratory complications. Our findings also indicate that certain lipids are elevated in response to injurious ventilation but not during LPV-NO, suggesting their potential utility as biomarkers for predicting postoperative outcomes in patients undergoing lung surgery. By further elucidating the complex biological processes that are involved in the development of lung injury and ARDS, we hope to move towards therapeutics that can target these pathways perioperatively in the future, further decreasing the incidence and severity of ARDS in lung resection patients.

## Figures and Tables

**Table 1:** Significantly upregulated lipids following OLV with IMV that had a fold change > 2.0. TG = triglyceride, DG = diglyceride, FFA = free fatty acid.

Name	Change	Fold Change (Log2)	Significance (Log10_10_)
**TG 18:2-18:2-20:4**	Increased	4.0288	2.3941
**DG 18:1-20:4**	Increased	2.7491	2.1928
**DG 16:0-20:4**	Increased	2.575	2.5863
**C18-2 LinoleoylCarnitine**	Increased	2.25	2.1294
**FFA 20:5**	Increased	2.1139	1.553

**Table 2:** Significantly upregulated lipids following OLV with LPV-HO that had a fold change > 2.0. LPE = lysophosphatidylethanolamine, FFA = free fatty acid, PS = phosphatidylserine, LPC = lysophosphatidylcholine, TG = triglyceride, PE = phosphatidylethanolamine.

Name	Change	Fold Change (log2)	Significance (Log10_10_)
**LPE 18:3**	Increased	2.5284	1.7269
**LPE 20:4**	Increased	2.4658	1.8035
**LPE 18:2**	Increased	2.4356	1.7149
**LPE 22:6**	Increased	2.3777	2.2529
**FFA 20:4**	Increased	2.2962	1.5721
**PS 38:5**	Increased	2.2747	2.1784
**LPC 22:6**	Increased	2.2624	3.7333
**TG 18:2-18:2-20:4**	Increased	2.2341	1.579
**FFA 20:5**	Increased	2.225	1.7884
**C18-2 LinoleoylCarnitine**	Increased	2.1522	2.3936
**LPC 18:3**	Increased	2.1233	2.7522
**PE 36:5**	Increased	2.0962	2.6673
**LPC 18:1**	Increased	2.0455	3.117

## Data Availability

The original contributions presented in this study are included in the article/Supplementary Materials. Further inquiries can be directed to the corresponding authors.

## Supplementary Materials

The following supporting information can be downloaded at: https://www.mdpi.com/article/10.3390/ijms27125219/s1.
