# Supplementary material for: Plasma Lipidomics Identify Pathways Linked to Acute Lung Injury in a Porcine One-Lung Ventilation Surgery Model
Source: Int J Mol Sci. 2026 Jun 9;27(12):5219. doi: 10.3390/ijms27125219 (PMC13299324; doi:10.3390/ijms27125219)
Supplement: Supplementary file 1 [file ijms-27-05219-s001.zip › ijms-4263615-supplementary.pdf]

Figure S1

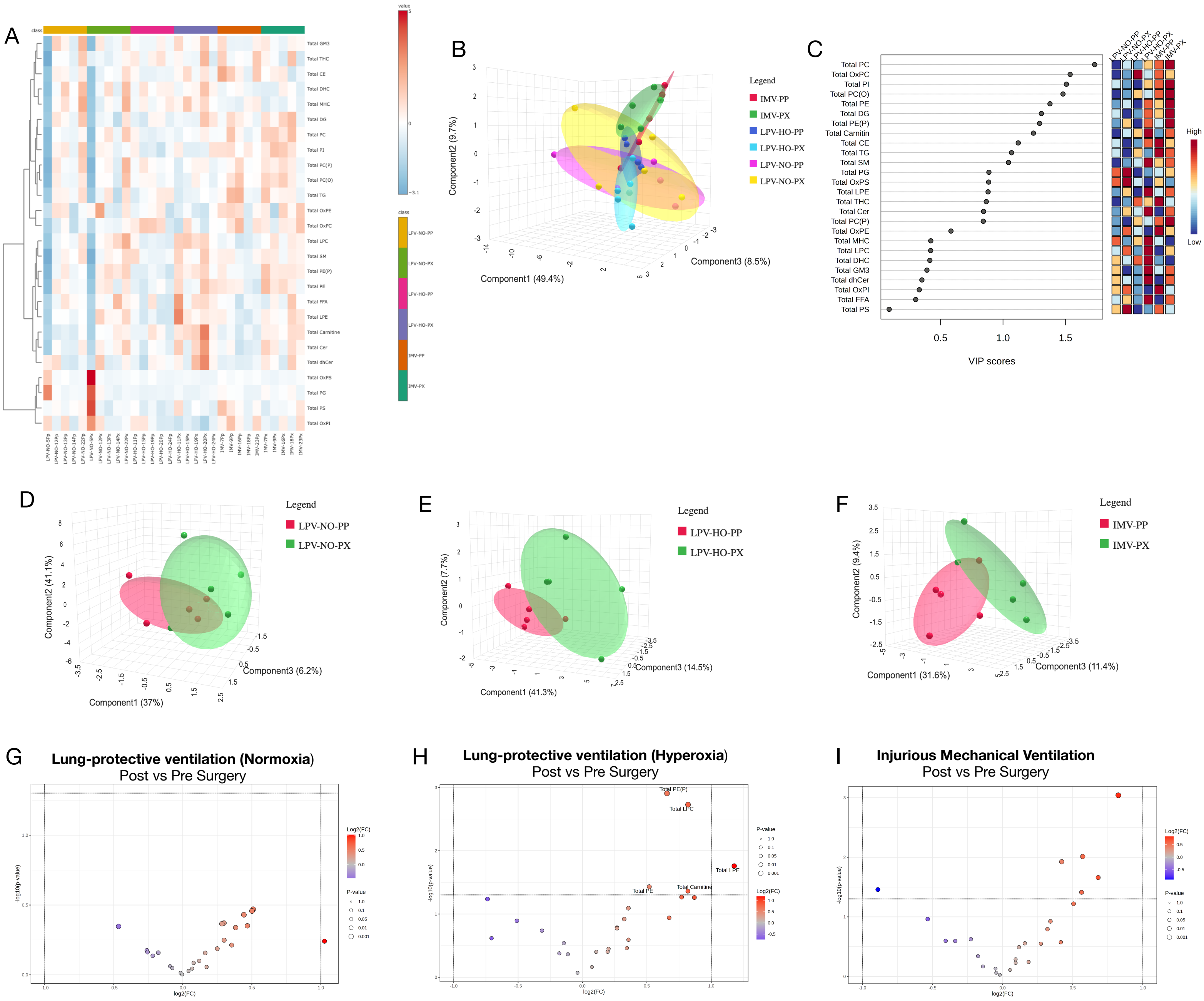

Figure S1. Total lipid profile displaying 26 lipid classes. (A) Unclustered heat map displaying blood plasma lipid abundance of all total lipid classes across all groups: LPV-NO, LPV-HO, and IMV. (B) PLSDA analysis displaying the overall lipid clustering covariances among all groups, where points positioned closer together indicates higher profile similarities, while distant points differ. (C) Variables with VIP > 1 (Variable Importance in Projection) are highly influential for the separation observed in PLSDA analysis, including total lipid classes such as: PC, OxPC, PI, PC(O), PE, DG, PE(P), Carnitine. (D) PLSDA analysis displaying the overall total lipid clustering covariances in among LPV-NO pre-surgery and post-surgery groups and (G) volcano plot comparing post vs pre, indicating no statistical significant changes. (E) PLSDA analysis displaying the overall total lipid clustering covariances in among LPV-HO pre-surgery and post-surgery groups and (H) volcano plot comparing post vs pre, indicating significant up-regulation of Total LPE. (F) PLSDA analysis displaying the overall total lipid clustering covariances in among IMV pre-surgery and post-surgery groups and (I) volcano plot comparing post vs pre, indicating no statistical significant changes. Volcano plot analysis were performed with FC=2, p≤0.05, non-parametric analysis Wilcoxon rank-sum test (also known as the Mann-Whitney U test) as the method for generating volcano plots when comparing two independent groups. Group legend: LPV-NO-PP = lung-protective ventilation with normoxia pre-OLV. LPV-NO-PX = lung-protective ventilation with normoxia post-OLV. LPV-HO-PP = lung-protective ventilation with hyperoxia pre-OLV. LPV-HO-PX = lung-protective ventilation with hyperoxia post-OLV. IMV-PP = injurious mechanical ventilation pre-OLV. IMV-PX = injurious mechanical ventilation post-OLV.

Table S1

Lipid class/subclass	Internal standard	μM/30 μL
SM	SM 12:0	0.5
PC	PC 13:0_13:0	0.5
PC(O)	PC 13:0_13:0	0.5
PC(P)	PC 13:0_13:0	0.5
LPC	LPC 13:0	0.5
LPC(O)	LPC 13:0	0.5
PE	PE 17:0_17:0	0.5
PE(O)	PE 17:0_17:0	0.5
PE(P)	PE 17:0_17:0	0.5
LPE	LPE 14:0	0.5
PI	PE 17:0_17:0	0.5
PS	PS 17:0_17:0	0.5
PG	PG 17:0_17:0	0.5
CE	CE 18:0 (*d6)*	20
DG	DG 15:0_15:0	5
TG	TG 17:0_17:0_17:0	5
FA	FA 15:0 (*d3*)	0.5

Table S1: Lipid internal standards and quantification used in this study.
